# Construction and validation of a machine learning model integrating ultrasound features and inflammatory markers (OVART-ML) for predicting ovarian torsion and ischemic necrosis risk in children

**DOI:** 10.3389/fped.2025.1717545

**Published:** 2025-12-04

**Authors:** Zhifei Zhao, Yubing Wang, Binyi Yang, Jiaxiang Tang, Jinbin Wang, Shujie Song, Yuezhen Zhang, Hongting Lu

**Affiliations:** 1 Department of Pediatric Surgery, Women and Children’s Hospital Affiliated to Qingdao University, Qingdao, China; 2 Department of Pediatric Surgery, Linyi People’s Hospital, Linyi, China

**Keywords:** ovarian torsion, machine learning, prediction model, SHapley additive Explanations (SHAP), pediatric surgery

## Abstract

**Objective:**

To construct a machine learning (ML) model (OVART-ML) using multimodal clinical data for predicting the risk of ovarian torsion (OT) and secondary ischemic necrosis (IN) in children and to identify key factors to assist clinical decision-making.

**Methods:**

A retrospective analysis was conducted on data (demographic characteristics, symptoms, ultrasonic findings, and laboratory indicators) of 112 children with ovarian space-occupying lesions admitted to Qingdao Women and Children's Hospital and Linyi People's Hospital between January 2012 and December 2024. After preprocessing (data standardization and LASSO feature selection), 11 ML algorithms [including Support Vector Machine [SVM], K-Nearest Neighbors [KNN], and Random Forest [RF]] were used to construct predictive models. Model performance was evaluated using indicators such as the Area Under the Curve (AUC), accuracy, and specificity. Key risk factors were identified using SHapley Additive exPlanations (SHAP).

**Results:**

Among 112 children, 60 (53.6%) developed OT and 23 (20.5%) developed IN. The SVM model exhibited the optimal performance: in the test set, its AUC was 0.911 [95% Confidence Interval (95% CI): 0.809–1.000], accuracy was 0.882, sensitivity was 0.900, and specificity was 0.857. SHAP analysis identified 8 key factors: the follicular edema ring sign, vomiting, pelvic effusion, eosinophil (EOS) count, white blood cell (WBC) count, hemoglobin (Hb) level, Neutrophil-to-Eosinophil Ratio (NER), and Systemic Immune-Inflammatory Index (SII). Among these, the follicular edema ring sign (mean |SHAP value| = 0.12) and EOS count (mean |SHAP value| = 0.08) had the highest predictive weights.

**Conclusion:**

This study developed an interpretable ML model that could accurately predict the risks of OT and IN in children. Key factors such as the follicular edema ring sign and vomiting provide important references for early diagnosis and intervention. This tool may assist clinicians in making timely surgical decisions to preserve ovarian function in children.

## 1 Introduction

Ovarian torsion (OT) is a common acute condition encountered in pediatric gynecology. OT has an acute onset and progresses rapidly; delayed diagnosis or misdiagnosis may lead to ovarian ischemic necrosis (IN) and loss of ovarian function, which affects long-term reproductive health (1, 2). The clinical manifestations are usually nonspecific and include acute abdominal pain (AAP), nausea, and vomiting. Currently, a definitive clinical diagnosis can only be confirmed by surgery (3), and early diagnosis relies solely on ultrasound, symptom assessment, and laboratory tests, which have limitations in sensitivity and specificity. For example, ultrasonic findings of early torsion are atypical, and symptoms such as abdominal pain are easily confused with gastrointestinal diseases (GIDs) [e.g., acute appendicitis (AA)] (4, 5).

Additionally, the search for reliable laboratory biomarkers to aid in diagnosis has remained a research focus. For instance, in a recent multicenter study, Delgado-Miguel et al. demonstrated that the neutrophil-to-lymphocyte ratio (NLR) serves as a valuable predictor for pediatric ovarian torsion (6). This finding highlights the critical role of the systemic inflammatory response in the pathophysiology of ovarian torsion.

However, a single inflammatory marker (e.g., NLR) exhibits inherent limitations in its predictive capacity (7, 8). OT and subsequent IN constitute a complex process involving local anatomy, systemic inflammation, and physiological reserve. Thus, integrating multi-dimensional data that encompasses more specific ultrasound features, a panel of inflammatory indices [e.g., eosinophil count, systemic immune-inflammation index (SII)], and patient demographics may facilitate the development of a more robust predictive tool.

Machine learning (ML) approaches are well-suited to address such complex, high-dimensional data. However, existing machine learning studies have not been extensively applied in the field of pediatric OT, and they commonly suffer from the “black-box” problem, a problem in which the model's decision-making process lacks transparency and hinders clinicians’ trust in and adoption of such models. This study was designed with two core objectives: the first is to develop and validate a machine learning model (OVART-ML) that incorporates ultrasound features and inflammatory markers for predicting the risk of pediatric OT and IN; the second is to utilize interpretable techniques such as SHAP to elucidate the key driving factors behind the model's decision-making, ultimately delivering an accurate and interpretable decision-support tool for clinicians.

## 2 Methods

**Figure 1:**
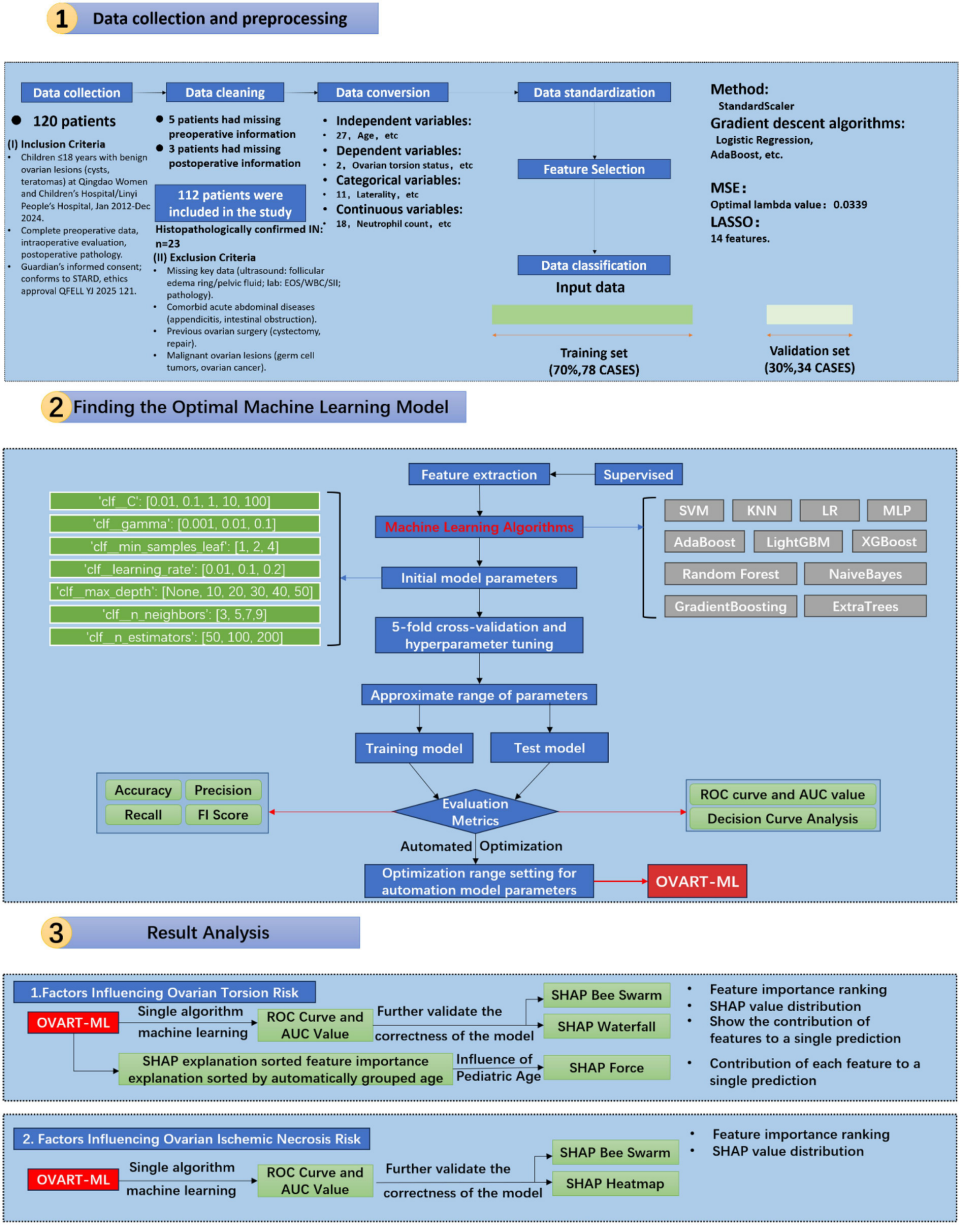
Flowchart of the methodology for predicting ovarian torsion and ischemic necrosis via machine learning.

This study focused on three main parts—data collection and preprocessing, construction of the optimal ML model, and result analysis—as shown in Figure 1.

### 2.1 Data collection and preprocessing

The clinical information of 120 children with ovarian space-occupying lesions admitted to Qingdao Women and Children's Hospital and Linyi People's Hospital from January 2012 to December 2024 was collected and de-identified to protect patient privacy. The data included the following: 4 demographic variables [age, height, weight, and body mass index (BMI)]; 4 clinical symptom variables (presence of vomiting, characteristics of abdominal pain, presence of abdominal tenderness, and presence of fever); 6 ultrasonic imaging features (which were assessed independently by at least two physicians blinded to the final diagnosis; laterality, maximum lesion diameter, presence of pelvic free fluid, follicular edema ring sign, echo characteristics, and Doppler flow signal); 11 laboratory parameters (white blood cell count [WBC], neutrophil count, lymphocyte count, monocyte count, eosinophil count [EOS], hemoglobin level [Hb], platelet count [PLT], and C-reactive protein [CRP]); derived indicators [such as the Systemic Immune-Inflammatory Index [SII = platelet × neutrophil/lymphocyte] and Neutrophil-to-Eosinophil Ratio [NER].Furthermore, intraoperative evaluations and pathological findings were documented to define the two primary endpoints of this study: OT and ovarian IN. These two endpoints were defined based on distinct diagnostic gold standards, with clear clinical and pathological differentiation: OT was confirmed by surgeons intraoperatively through direct visual inspection, adhering to diagnostic criteria that included typical intraoperative findings such as rotation of the ovary and its vascular pedicle, ovarian congestion and edema, and color changes (9); Ovarian IN was strictly defined as cases with pathologically confirmed irreversible cellular necrosis (e.g., pyknosis, karyorrhexis, cytoplasmic eosinophilia) via postoperative histopathology (10), and was restricted to patients who underwent oophorectomy due to intraoperative assessment of ovarian non-viability and for whom pathological reports were available. Correspondingly, the ovarian preservation group was defined as cases where the ovary was assessed to be viable intraoperatively, successful detorsion was performed, and no oophorectomy was conducted. These definitions ensured the objectivity, verifiability, and mutual exclusivity of the endpoints, providing a reliable basis for subsequent analyses.

This study was approved by the Ethics Committee of Qingdao Women and Children's Hospital Affiliated to Qingdao University (approval no.: QFELLYJ2025-121). Given the retrospective design of this study and the fact that all data had been de-identified prior to analysis, the ethics committee approved a waiver of informed consent from patients. This study was conducted in accordance with the STARD reporting guidelines (11) and complied with the ethical principles outlined in the Declaration of Helsinki.

#### 2.1.1 Data cleaning

To ensure data integrity, incomplete datasets (owing to missing key patient information or subsequent data caused by human error) were excluded from the analysis based on the study objectives. In the cohort, 5 patients lacked complete laboratory test information, and 3 patients lacked ultrasonic information. After applying these criteria, data from 112 patients were finally included in this study. The exclusion criteria are shown in Figure 1.

#### 2.1.2 Data conversion

The independent variables included 4 demographic variables, 4 clinical symptom variables, 6 ultrasonic imaging features, and 13 laboratory parameters and derived indicators, totaling 27 variables. The dependent variables included OT status and ovarian IN status, totaling 2 variables. Categorical variables were numerically formatted using the pandas module in Python 3.8.

#### 2.1.3 Data classification

To meet ML requirements, the dataset was divided into a training set (78 patients, 70%) and a validation set (34 patients, 30%). Iterative division improved the model accuracy training, as the dataset was reused through appropriate functions.

#### 2.1.4 Data standardization

For distance-based algorithms and algorithms requiring covariance matrix calculations [e.g., Logistic Regression (LR) and AdaBoost], data standardization improves model performance, prevents gradient issues, and balances feature dimensions. Continuous data were converted to a standard normal distribution [mean = 0, standard deviation (SD) = 1] using the StandardScaler function in Python 3.8.

#### 2.1.5 Feature selection

To avoid overfitting and improve model efficiency, the Least Absolute Shrinkage and Selection Operator (LASSO) regression was used for feature selection. This method penalizes the absolute value of the regression coefficients and can effectively drive the coefficients of less important features to zero. The optimal λ value was determined by minimizing the Mean Squared Error (MSE), and 14 variables were finally retained.

### 2.2 Machine learning-based model for predicting ovarian torsion and ischemic necrosis risk (OVART-ML)

#### 2.2.1 Construction of ML models

Custom programs were written to construct an ML model library, including Support Vector Machine (SVM), K-Nearest Neighbors (KNN), Random Forest (RF), Extra Trees (ET), Extreme Gradient Boosting (XGBoost), Light Gradient Boosting Machine (LightGBM), Gradient Boosting (GB), AdaBoost, Logistic Regression (LR), Naive Bayes (NB), and Multi-Layer Perceptron (MLP).

There are several types of ML algorithms, each with unique characteristics. The 11 algorithms used in this study have been proven by numerous mathematicians and medical researchers to be classic models for predicting outcomes of classification problems (12–14); the effectiveness and correctness of these models have also been verified by many scholars. However, in previous studies, these models were often used alone for outcome prediction, which has some limitations: (1) the prediction stability of the software varies greatly, and (2) the models/algorithms lack universality and are not suitable for predicting the risks of OT and IN in pediatric patients. To address the above issues and improve the stability of the developed ML model, this study developed the OVART-ML model using the above 11 ML algorithms based on the Python 3.8 coding platform, which significantly improved the accuracy and stability of patient outcome prediction.

#### 2.2.2 Initialization of model parameters

The Pipeline function was used to efficiently standardize data and initialize model parameters. The key parameters included clf_C, clf_gamma, clf_min_Samples_leaf, clf_learning_rate, clf_max_depth, clf_n_neighbors, and clf_n_estimators. Hyperparameter optimization and model training are detailed in the Supplementary File S1.

#### 2.2.3 5-fold cross-validation (5-FCV)

To maximize the utility of the collected data, a 5-FCV algorithm was adopted. The training set was divided into 5 subsamples. One subsample was retained for validation, and the remaining 4 were used for training. This process was repeated 5 times; the average result was used as the final prediction. This method ensured that each subsample was used for validation once, and the robustness of the model in training and validation was enhanced using randomly generated subsamples.

#### 2.2.4 Evaluation criteria

To comprehensively evaluate the predictive performance of the model, the following metrics were employed: receiver operating characteristic curve (ROC curve), area under the curve (AUC), calibration slope, calibration intercept, decision curve analysis (DCA), accuracy, and precision. The optimal probability threshold for converting the model's output continuous probability into binary classification results (e.g., “torsion/non-torsion”) was determined exclusively on the training set. Specifically, Youden's index was used to identify the threshold that maximizes the sum of sensitivity and specificity; this threshold was then fixed and directly applied to the test set to calculate classification metrics such as sensitivity and specificity. This approach ensured no information leakage, enabling unbiased estimation of model performance. Additionally, to assess and mitigate the risk of overfitting, all models were subjected to internal validation using the Bootstrap method with 1000 repeated resamplings (15).

### 2.3 Interpretability analysis

A custom data-postprocessing program was created using Python 3.8. SHAP was used to enhance the clinical interpretability of the model. SHAP values quantify the contribution of each feature to the individual prediction of a specific case and explain how the model arrives at its results. The results included the following: SHAP summary plots, waterfall plots, dependence plots, and interaction plots for visualizing the impact and relationships of the top features.

## 3 Results

### 3.1 Baseline characteristics of children

In total, 112 children with ovarian space-occupying lesions were included in the final analysis. Based on surgical and pathological results, 23 children (20.5%) were assigned to the necrosis group [undergoing oophorectomy (OPE)] and 89 patients (79.5%) to the preservation group. Patient baseline characteristics are summarized in Table 1. Significant differences were observed between the two groups in terms of the incidence of vomiting, ultrasonic detection of pelvic free fluid, EOS, and SII.

**Table 1:** Baseline characteristics of children with ovarian torsion.

Feature variables	Total (*n* = 112)	Preservation group (*n* = 89)	Necrosis group (*n* = 23)
Demographic characteristics			
Age (months), median (IQR)	111.0 (68.0, 144.0)	115 (75.5, 144.0)	84.0 (14.0, 156.0)
Clinical symptoms, *n* (%)			
Vomiting	48 (42.9%)	36 (40.4%)	12 (52.2%)
Abdominal tenderness	76 (67.9%)	61 (68.5%)	15 (65.2%)
Peritoneal irritation sign	8 (7.1%)	5 (5.6%)	3 (13.0%)
Ultrasonic imaging features, *n* (%)			
Pelvic free fluid	54 (48.2%)	41 (46.1%)	13 (56.5%)
Follicular edema ring sign	45 (40.2%)	28 (41.6%)	8 (34.8%)
Vascular signal	14 (12.5%)	7 (7.9%)	7 (30.4%)
Laboratory tests, median (IQR)			
White blood cell count (WBC, ×10^9^ /L)	8.85 (6.48, 12.19)	8.33 (6.37, 11.65)	12.08 (7.82, 15.44)
Eosinophil count (EOS, ×10^9^ /L)	0.08 (0.02, 0.13)	0.09 (0.02, 0.16)	0.03 (0.01, 0.05)
Hemoglobin (Hb, g/L)	130.0 (122.0, 137.5)	130.5 (123.0, 136.75)	125 (110, 136.5)
Platelet count (PLT, ×10^9^ /L)	292.0 (248.5, 336.5)	292.0 (240.0, 337.0)	306.0 (262.5, 376)
C-Reactive protein (CRP, mg/L)	0.40 (0.20, 4.93)	0.27 (0.20, 1.95)	18.17 (0.75, 49.02)
Derived indicators, median (IQR)			
Systemic immune-inflammatory index (SII)	707.6 (372.7, 1,511.1)	573.9 (354.0, 1,332.8)	1,524.2 (1,130.4, 2,279.1)
Neutrophil-to-lymphocyte ratio (NLR)	2.88 (1.40, 5.69)	2.09 (1.29, 4.49)	5.28 (3.20, 7.98)
Neutrophil-to-eosinophil ratio (NER)	51.22 (23.03, 258.4)	49.25 (18.2, 264.8)	307.3 (182.0, 454.5)

### 3.2 Results of data preprocessing and feature selection

LASSO regression was used for feature selection. The optimal *λ* value determined by minimizing the MSE was 0.0339, and 14 variables were retained from 27 independent variables. Thereafter, combined with a preliminary analysis of the feature importance of the 11 algorithms and clinical relevance, 8 core modeling features were further screened: vomiting, follicular edema ring sign, pelvic effusion, EOS, WBC, Hb, NER, and SII (Supplementary Figures 1A,B,N for the screening basis).

### 3.3 Model performance and selection

#### 3.3.1 Multi-Dimensional comparison of multi-model performance

Using the aforementioned 8 variables, we developed and systematically compared the predictive performance of 11 machine learning models for OT, with 60 events and an event per variable (EPV) of 7.5; results are presented in Table 2. The SVM model exhibited the most balanced performance on the test set: accuracy of 0.882, AUC of 0.911 [95% confidence interval (CI): 0.809–1.000], sensitivity of 0.900, specificity of 0.857, and F1-score of 0.900. In contrast, although some ensemble models demonstrated “perfect” performance on the training set, their performance declined noticeably on the test set—for instance, the AUC of GB on the test set dropped from 0.98990 to 0.823827—indicating a tendency toward overfitting.

**Table 2:** Performance evaluation of the OVART-ML model.

Model name	Accuracy	AUC(95%CI)	Brier score	Calibration slope linear	Calibration intercept linear	Average calibration slope isonotic	Sensitivity (95% CI)	Specificity (95% CI)	PPV (95% CI)	NPV (95% CI)	Precision	Recall	F1	Threshold	Task
LR	0.885	0.938 (0.8861–0.9902)					0.8750 (0.7725–0.9775)	0.8947 (0.7972–0.9923)	0.8974 (0.8022–0.9927)	0.8718 (0.7669–0.9767)	0.897	0.875	0.886	0.461	Train
LR	0.912	0.889 (0.7441–1.0000)	0.095	0.928	0.073	0.426	0.9000 (0.7685–1.0000)	0.9286 (0.7937–1.0000)	0.9474 (0.8470–1.0000)	0.8667 (0.6946–1.0000)	0.947	0.900	0.923	0.541	Test
NB	0.859	0.911 (0.8468–0.9743)					0.8750 (0.7725–0.9775)	0.8421 (0.7262–0.9580)	0.8537 (0.7455–0.9618)	0.8649 (0.7547–0.9750)	0.854	0.875	0.864	0.402	Train
NB	0.912	0.886 (0.7417–1.0000)	0.090	0.855	0.109	0.392	0.9000 (0.7685–1.0000)	0.9286 (0.7937–1.0000)	0.9474 (0.8470–1.0000)	0.8667 (0.6946–1.0000)	0.947	0.900	0.923	0.687	Test
SVM	0.910	0.953 (0.9092–0.9974)					0.9000 (0.8070–0.9930)	0.9211 (0.8353–1.0000)	0.9231 (0.8394–1.0000)	0.8974 (0.8022–0.9927)	0.923	0.900	0.911	0.575	Train
SVM	0.882	0.911 (0.8090–1.0000)	0.120	1.118	−0.077	1.781	0.9000 (0.8070–0.9930)	0.8571 (0.6738–1.0000)	0.9000 (0.7685–1.0000)	0.8571 (0.6738–1.0000)	0.900	0.900	0.900	0.669	Test
KNN	0.872	0.935 (0.8871–0.9832)					0.9250 (0.8434–1.0000)	0.8158 (0.6925–0.9390)	0.8409 (0.7328–0.9490)	0.9118 (0.8164–1.0000)	0.841	0.925	0.881	0.400	Train
KNN	0.912	0.912 (0.7954–1.0000)	0.095	1.001	0.068	0.734	0.9000 (0.7685–1.0000)	0.9286 (0.7937–1.0000)	0.9474 (0.8470–1.0000)	0.8667 (0.6946–1.0000)	0.947	0.900	0.923	0.600	Test
RF	1.000	1.0000 (1.0000–1.0000)					1.0000 (1.0000–1.0000)	1.0000 (1.0000–1.0000)	1.0000 (1.0000–1.0000)	1.0000 (1.0000–1.0000)	1.000	1.000	1.000	0.600	Train
RF	0.794	0.814 (0.6568–0.9718)	0.172	0.847	0.053	0.578	0.7500 (0.5602–0.9398)	0.8571 (0.6738–1.0000)	0.8824 (0.7292–1.0000)	0.7059 (0.4893–0.9225)	0.882	0.750	0.811	0.800	Test
ET	1.000	1.000 (1.0000–1.0000)					1.0000 (1.0000–1.0000)	1.0000 (1.0000–1.0000)	1.0000 (1.0000–1.0000)	1.0000 (1.0000–1.0000)	1.000	1.000	1.000	1.000	Train
ET	0.912	0.930 (0.8361–1.0000)	0.094	0.986	0.018	0.775	0.9500 (0.8545–1.0000)	0.8571 (0.6738–1.0000)	0.9048 (0.7792–1.0000)	0.9231 (0.7782–1.0000)	0.905	0.950	0.927	0.500	Test
XGB	1.000	1.000 (1.0000–1.0000)					1.0000 (1.0000–1.0000)	1.0000 (1.0000–1.0000)	1.0000 (1.0000–1.0000)	1.0000 (1.0000–1.0000)	1.000	1.000	1.000	0.604	Train
XGB	0.912	0.887 (0.7546–1.0000)	0.109	1.055	−0.013	0.670	0.9000 (0.7685–1.0000)	0.9286 (0.7937–1.0000)	0.9474 (0.8470–1.0000)	0.8667 (0.6946–1.0000)	0.947	0.950	0.923	0.607	Test
LGB	0.885	0.935 (0.8822–0.9876)					0.8250 (0.7072–0.9428)	0.9474 (0.8764–1.0000)	0.9429 (0.8660–1.0000)	0.8372 (0.7269–0.9476)	0.943	0.825	0.880	0.540	Train
LGB	0.912	0.904 (0.7814–1.0000)	0.129	1.758	−0.328	1.420	0.9000 (0.7685–1.0000)	0.9286 (0.7937–1.0000)	0.9474 (0.8470–1.0000)	0.8667 (0.6946–1.0000)	0.947	0.900	0.923	0.540	Test
GB	0.962	0.990 (0.9739–1.0000)					0.9750 (0.9266–1.0000)	0.9474 (0.8764–1.0000)	0.9512 (0.8853–1.0000)	0.9730 (0.9207–1.0000)	0.951	0.975	0.963	0.511	Train
GB	0.824	0.827 (0.6753–0.9783)	0.158	1.246	−0.121	1.348	0.9000 (0.7685–1.0000)	0.7143 (0.4776–0.9509)	0.8182 (0.6570–0.9794)	0.8333 (0.6225–1.0000)	0.818	0.900	0.857	0.379	Test
AdaBoost	0.949	0.985 (0.9641–1.0000)					0.9500 (0.8825–1.0000)	0.9474 (0.8764–1.0000)	0.9500 (0.8825–1.0000)	0.9474 (0.8764–1.0000)	0.950	0.950	0.950	0.506	Train
AdaBoost	0.853	0.821 (0.6521–0.9908)	0.211	0.790	0.131	2.394	0.8500 (0.6935–1.0000)	0.8571 (0.6738–1.0000)	0.8947 (0.7567–1.0000)	0.8000 (0.5976–1.0000)	0.895	0.850	0.872	0.478	Test
MLP	0.885	0.924 (0.8647–0.9840)					0.8250 (0.7072–0.9428)	0.9474 (0.8764–1.0000)	0.9429 (0.8660–1.0000)	0.8372 (0.7269–0.9476)	0.943	0.825	0.880	0.505	Train
MLP	0.912	0.886 (0.7519–1.0000)	0.117	1.200	−0.042	0.746	0.9000 (0.7685–1.0000)	0.9286(0.7937–1.0000)	0.9474(0.8470–1.0000)	0.8667(0.6946–1.0000)	0.947	0.900	0.923	0.451	Test

To assess model optimism bias, we further conducted internal validation using the Bootstrap method with 1000 repetitions. Results showed that all models exhibited only “mild” overfitting. Specifically, the SVM model had an original AUC of 0.911 on the test set, with an optimism-corrected value of only 0.003, resulting in a corrected AUC of 0.907. It's Brier score increased slightly from 0.120 to 0.124, demonstrating good generalization ability and minimal optimism bias. (See Supplementary Table S1 for complete results.).

#### 3.3.2 Feature ablation analysis and supplementary validation

To validate the incremental value of the “ultrasound-inflammation-clinical” multimodal strategy, we developed two types of unimodal baseline models based on SVM: an ultrasound unimodal model (incorporating 6 imaging features) and a serological unimodal model (including 5 inflammatory indices: EOS, WBC, Hb, NER, SII). All other procedures—including the 7:3 data split, 5-fold grid search, and evaluation criteria—were consistent with those of the full-feature model.

Results showed that the full-feature model (AUC = 0.911) outperformed both the ultrasound unimodal model (AUC = 0.898) and the serological unimodal model (AUC = 0.729). ROC curves are provided in the (Supplementary Figure S2), confirming that multimodal fusion significantly enhances predictive performance. A single data source, by contrast, cannot fully capture the complex pathophysiological characteristics of OT.

#### 3.3.3 Model calibration, clinical net benefit, and decision threshold

**Figure 2:**
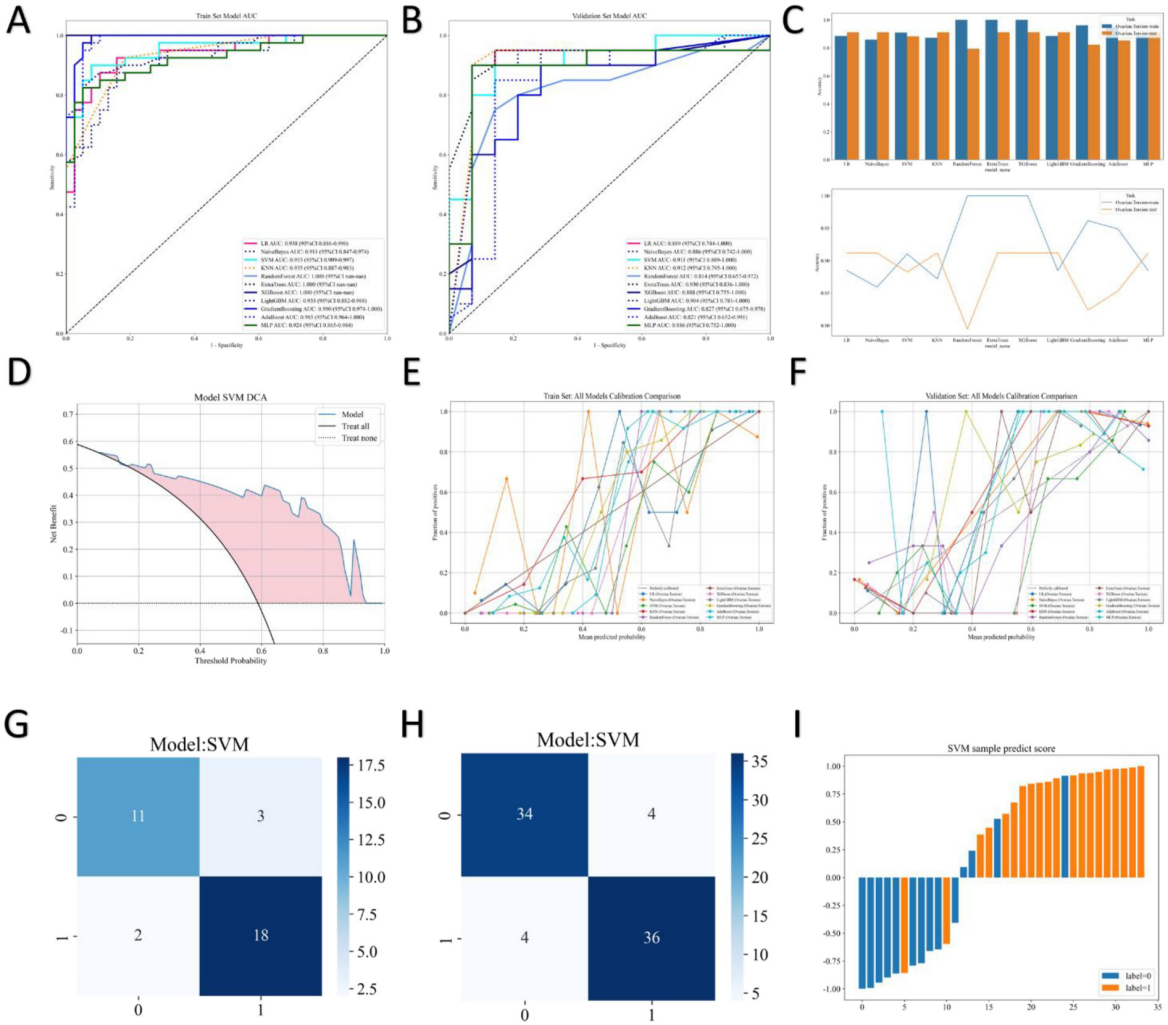
ROC curves for multiple models in the training set. **(A)** ROC curves for the same models in the validation set. **(B)** Model performance summary. **(C)** Decision Curve Analysis (DCA) for SVM: Evaluates the clinical net benefit of using the SVM model across a range of threshold probabilities. **(D)** Calibration in the training set. **(E)** Calibration in the validation set. **(F)** Confusion matrices for SVM In the training set. **(G)** Confusion matrices for SVM In the validation set. **(H)** SVM sample prediction scores. **(I)**.

Calibration curves demonstrated that the predicted probabilities of the SVM model were highly consistent with the actual occurrence probabilities in the validation set, and the curve was closely aligned with the 45° diagonal line. In contrast, models such as random forest and XGBoost exhibited obvious calibration offsets (Figure 2F).

Decision curve analysis (DCA) further indicated that within the threshold probability range of 0.1–0.8, the clinical net benefit of the SVM model was consistently higher than that of the “treat-all” or “treat-none” strategies. This advantage was particularly prominent in the 0.3–0.7 interval (Figure 2D), supporting its practical value in clinical decision-making.

Based on Youden's J (Youden's index), the optimal classification threshold was determined to be 0.669. At this threshold, the model achieved a sensitivity of 0.900, a specificity of 0.857, and a Youden's J of 0.757 on the test set, effectively balancing the risks of missed diagnosis and misdiagnosis (Table 3a). The confusion matrix showed 36 true positives, 34 true negatives, 4 false positives, and 4 false negatives in the test set (Figure 2H). The distribution of predicted scores also revealed high discriminability between the two groups of samples with minimal overlap (Figure 2I).

**Table 3:** Threshold-strategy analysis for ovarian torsion prediction using the SVM model.

(a) Performance metrics at different probability thresholds											
Row	Threshold	Sensitivity	Specificity	PPV	NPV	Accuracy	F1-Score	TP	FP	TN	FN
0	0.100	1.000	0.214	0.645	1.000	0.676	0.784	588	324	88	0
1	0.200	0.950	0.500	0.731	0.875	0.765	0.826	558	206	206	30
2	0.300	0.900	0.643	0.783	0.818	0.794	0.837	529	148	264	59
3	0.400	0.900	0.714	0.818	0.833	0.824	0.857	529	118	294	59
4	0.500	0.900	0.714	0.818	0.833	0.824	0.857	529	118	294	59
5	0.600	0.900	0.786	0.857	0.846	0.853	0.878	529	89	323	59
6	0.669	0.900	0.857	0.900	0.857	0.882	0.900	529	59	353	59
7	0.700	0.800	0.857	0.889	0.750	0.824	0.842	470	59	353	118
8	0.800	0.700	0.929	0.933	0.684	0.794	0.800	411	30	382	177
9	0.900	0.400	1.000	1.000	0.538	0.647	0.571	235	0	412	353
(b) Clinical decision implications for different threshold ranges											
Threshold range				Clinical strategy				Clinical implication			
Very Low (e.g., 0.1–0.2)				High-sensitivity rule-out				Maximizes sensitivity to exclude disease (e.g., in emergency triage), ensuring very low miss rate.			
Low (e.g., 0.3)				Liberal strategy				Prioritizes high sensitivity, suitable for screening high-risk populations.			
Moderate (e.g., 0.4–0.6)				Balanced/default strategy				Suitable for general diagnostic scenarios.			
0.669				Youden's index-optimized threshold				Best statistical trade-off between sensitivity and specificity, balancing miss and misdiagnosis risks.			
High (e.g., 0.7–0.8)				Strict strategy				Prioritizes high specificity, suitable for confirmatory testing or resource-limited settings.			
Very high (e.g., 0.9)				High-specificity rule-in				Maximizes specificity to confirm disease (e.g., for pre-operative asse			

#### 3.3.4 Conclusion on optimal model selection

Based on a multidimensional analysis of discriminative efficacy (including metrics such as AUC and accuracy), generalization ability (consistency of training–test set performance), calibration (matching between predicted and actual probabilities), and clinical net benefit (DCA results), the SVM model showed the best performance in predicting OT risk in children: it had good case discrimination efficacy (test-set AUC = 0.911), accurately calibrated the predicted probability, and provided reliable NB for clinical decision-making. Therefore, this model was selected as the final predictive model for this study.

### 3.4 SHAP feature analysis for ovarian torsion prediction

#### 3.4.1 Factor importance ranking and global interpretation

**Figure 3:**
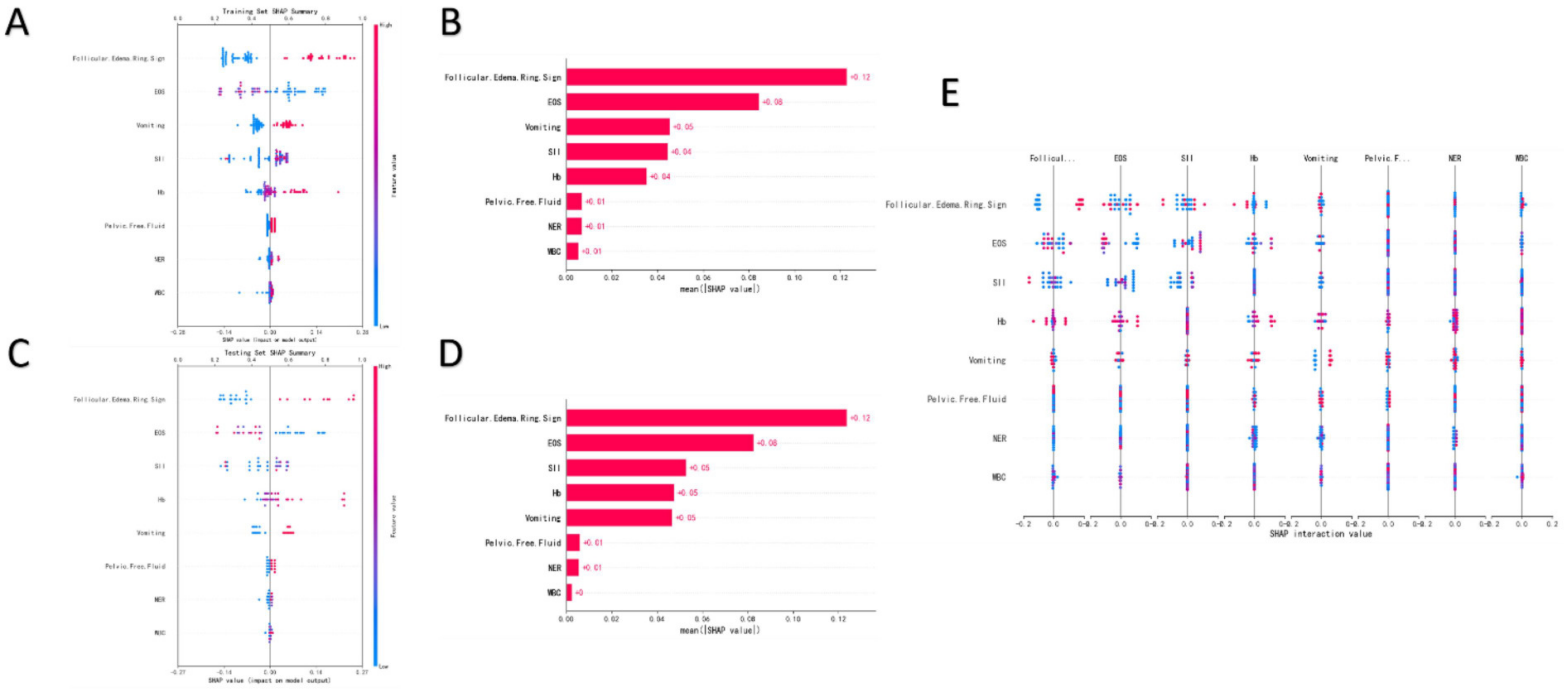
SHAP summary plot for the training set. **(A)** Feature importance bar plot for the training set. **(B)** SHAP summary plot for the testing set. **(C)** Feature importance bar plot for the testing set. **(D)** SHAP interaction value plot. **(E)**.

Comparative analysis of SHAP summary plots (Figures 3A,C) and feature importance bar plots (Figures 3B,D) revealed highly consistent ranking of risk factors by the model in both the training and test sets. Among these features, the follicular edema ring sign exhibited the highest mean absolute SHAP value (≈0.12 in the training set, with a consistent trend in the test set), which was significantly higher than that of other variables. This indicates that this ultrasonic sign is the most critical feature driving the prediction of OT.

It was followed by EOS, vomiting, SII, and Hb, with their mean absolute SHAP values ranging from 0.05 to 0.08; these constitute secondary important predictors. The high consistency of this ranking between the training and test sets confirms that the model has good generalization ability and stability in identifying core risk factors.

Notably, the SHAP value distribution of the follicular edema ring sign showed a clear directionality: in the presence of this sign (feature value = 1), most SHAP values were positive with large absolute values, which significantly increased the model's predicted probability of “OT”. Conversely, in the absence of this sign, SHAP values were close to zero or negative, reducing the predicted risk of torsion. This finding is highly consistent with clinical experience—the follicular edema ring sign is a specific ultrasonic manifestation of ovarian congestion and edema caused by impaired venous return in the early stage of OT, and it has high diagnostic specificity and value for early identification.

#### 3.4.2 Local interpretation of single-sample prediction: SHAP waterfall plot and force plot

**Figure 4:**
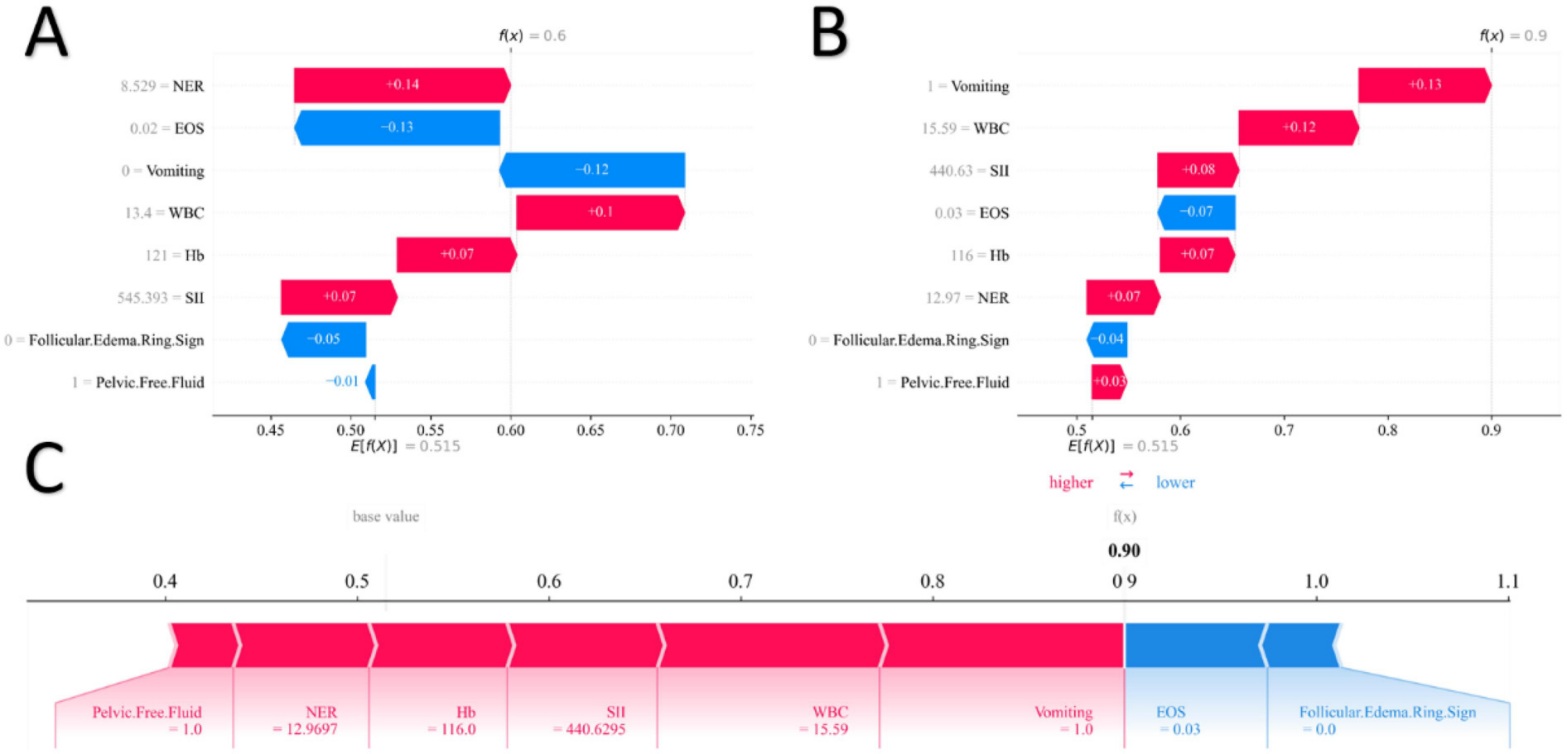
SHAP waterfall plots. **(A,B)** SHAP Force plot. **(C)**.

To further clarify the model's decision logic at the individual level, this study used SHAP waterfall plots to provide local interpretations of typical positive samples. Taking one surgically confirmed OT patient from the training set as an example (Figure 4A), the baseline predicted probability was 0.515, and the final output probability was 0.60. Among the features, vomiting (+0.12) and elevated white blood cell count (WBC = 13.4 × 10^9^ /L, + 0.10)—as typical manifestations of inflammation and peritoneal irritation—significantly increased the predicted risk. In contrast, decreased eosinophil count (EOS = 0.02 × 10^9^ /L, −0.13) exerted a negative contribution, which may reflect immune cell redistribution induced by ischemia-reperfusion injury after torsion. After the comprehensive action of multiple features, the model accurately output a high-risk prediction, consistent with the clinical outcome.

In another high-risk sample from the test set (Figure 4B), although the follicular edema ring sign was absent (−0.04), strongly positive features such as vomiting (+0.13), elevated WBC (15.59 × 10^9^ /L, +0.12), decreased hemoglobin (Hb = 116 g/L, + 0.07), and elevated neutrophil-to-eosinophil ratio (NER = 12.97, +0.07) collectively dominated the prediction. The final probability reached 0.90, which was highly consistent with the postoperative diagnosis of “OT with partial ischemia”. Notably, although the SII (SII = 440.63) showed a negative contribution (−0.08), its absolute value was small. Moreover, in the clinical context, this SII value was still higher than the reference range for healthy children, suggesting that its predictive role requires comprehensive judgment in combination with other features. SHAP force plots (Figure 4C) further visually demonstrated the dynamic balance between red (risk-promoting) and blue (risk-inhibiting) features, highlighting the key role of the “multi-factor superposition amplification effect” in individual risk assessment.

The above local interpretations not only revealed the synergistic mechanism across the three dimensions of “symptoms–inflammatory indices–ultrasonic features” but also verified the consistency in the model's interpretability between the training and test sets. This provides a quantifiable decision-support tool for clinically achieving “precision risk stratification for individual cases”.

#### 3.4.3 Feature interaction analysis

**Figure 5:**
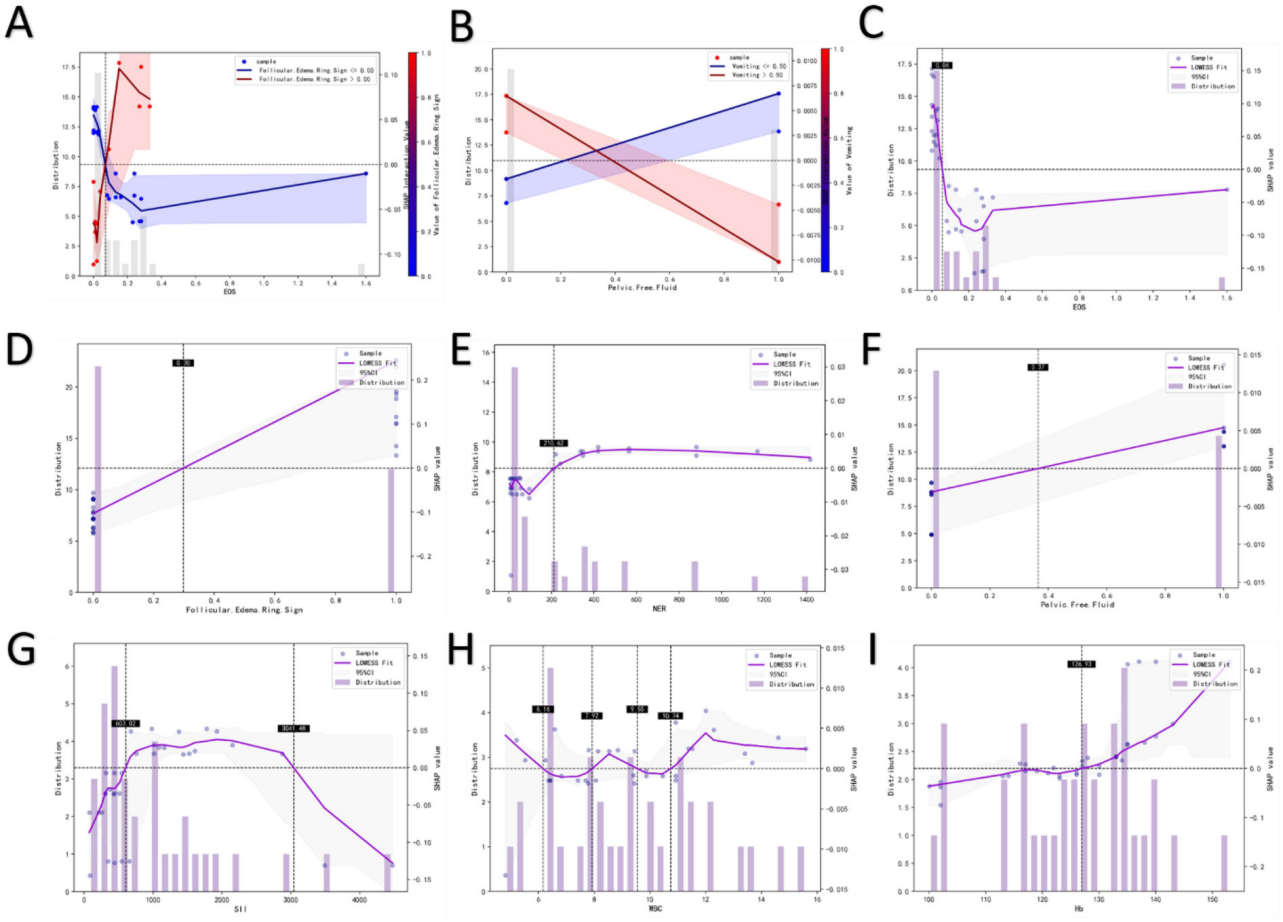
Bivariate SHAP dependence plots.**(A,B)** Univariate SHAP dependence plots **(C–I)**: EOS **(C)**, Follicular Edema Ring Sign **(D)**, NER **(E)**, Pelvic Free Fluid **(F)**, SII **(G)**,WBC **(H)**, Hb **(I)**.

SHAP interaction plots further revealed the nonlinear synergistic relationships between key features. First, in the interaction analysis of the follicular edema ring sign and EOS (Figure 5A), when the follicular edema ring sign was present (red curve), elevated EOS significantly increased the predicted risk of torsion. In contrast, when this sign was absent (blue curve), the impact of EOS on prediction was greatly weakened—even plateauing when EOS > 0.2 × 10^9^ /L. This indicates that the predictive value of EOS is highly dependent on the presence of direct ultrasonic signs, and their synergy can more accurately reflect the pathological process of “ovarian structural changes + systemic inflammatory response”.

Second, the interaction between vomiting and pelvic free fluid (Figure 5B) showed that pelvic fluid accumulation significantly increased the predicted risk of torsion only when vomiting was present. Without vomiting, the predictive efficacy of pelvic fluid accumulation decreased markedly. This pattern aligns with the clinical phenotype logic of “OT → peritoneal irritation (vomiting) + abdominal effusion (fluid accumulation)”, emphasizing the importance of combined interpretation of symptoms and indirect imaging signs for improving diagnostic confidence.

The above interaction effects demonstrate that the model does not simply sum the contributions of individual variables, but rather effectively captures the inherent correlations between clinical indicators. This enables more complex decision-making that aligns with pathophysiological mechanisms.

#### 3.4.4 Marginal effect of single features

SHAP dependence plots further quantified the independent impact trends of individual features. For EOS, within the range of <0.2 × 10^9^ /L, SHAP values decreased rapidly as EOS increased—indicating that low EOS levels have significant negative predictive value for OT. For NER, when <200, it showed a strong positive correlation with torsion risk, reflecting the close association between a neutrophil-dominant state and the inflammatory response of OT.

For SII, torsion risk increased with SII values when <1,000; however, SHAP values decreased in the opposite direction when SII > 3,000, which may be interfered by confounding factors such as severe infection. For WBC, a non-monotonic relationship was observed within the normal range (6–10 × 10^9^ /L); when WBC > 12 × 10^9^ /L, its predictive efficacy tended to saturate, suggesting limited specificity. For Hb, SHAP values increased as Hb levels rose when <130 g/L, supporting the hypothesis that “OT leads to local ischemia/occult blood loss”.

Regarding ultrasonic features, SHAP dependence plots for the follicular edema ring sign (Figure 5D) and pelvic free fluid (Figure 5F) both showed: SHAP values increased significantly when these features were present and remained low when absent. This reaffirms their core predictive roles as direct and indirect imaging signs, respectively.

### 3.5 SHAP feature analysis for ovarian preservation prediction

#### 3.5.1 Model performance basis: discriminative efficacy of SVM

**Figure 6:**
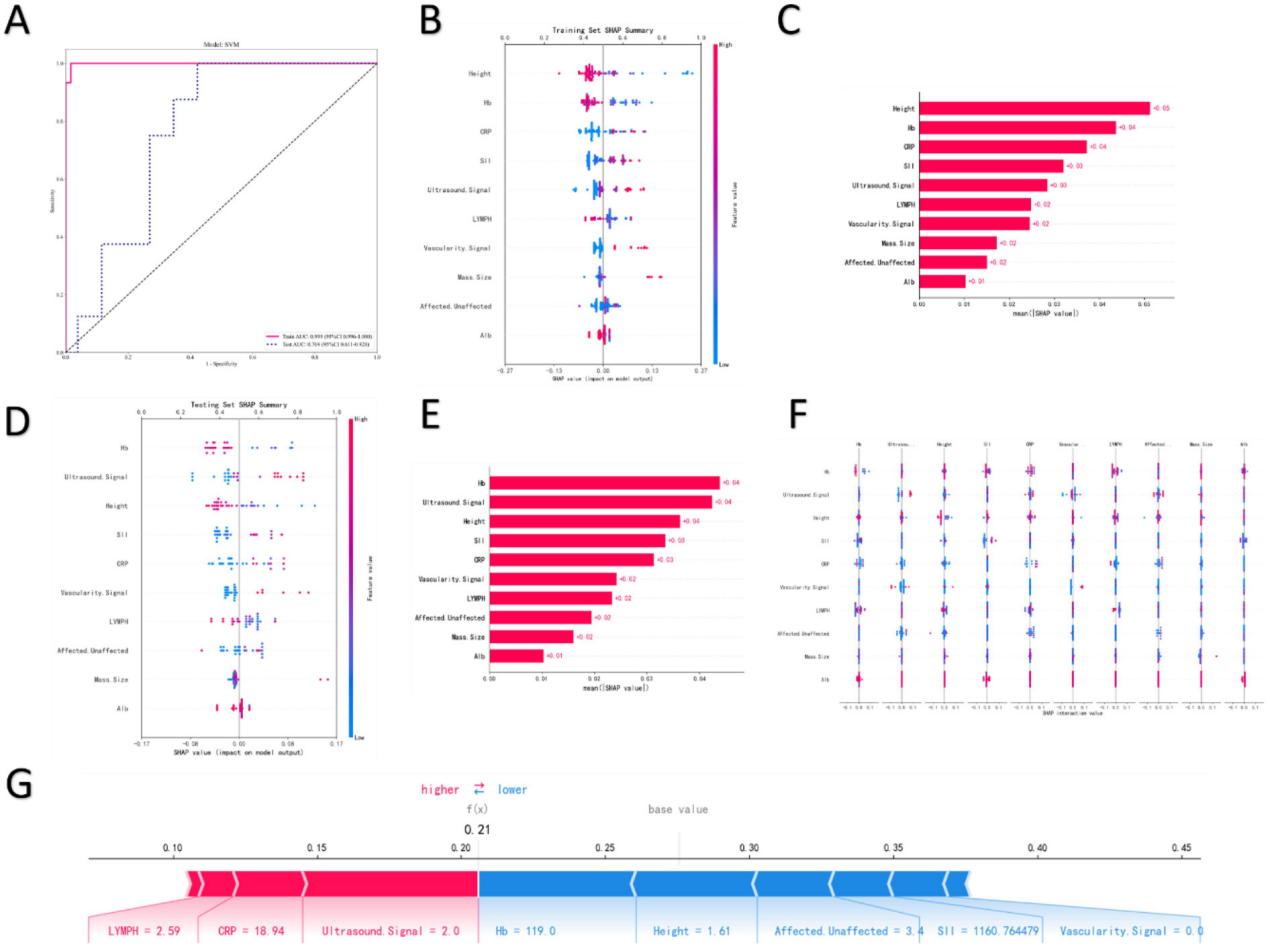
ROC curve for SVM. **(A)** SHAP summary plot for the training set. **(B)** Feature importance bar plot for the training set. **(C)** SHAP summary plot for the testing set. **(D)** Feature importance bar plot for the testing set. **(E)** SHAP interaction value plot. **(F)** SHAP force plot for an individual sample. **(G)**.

SVM was used to construct an ovarian preservation prediction model. The results of the ROC curve (Figure 6A) showed that the training-set AUC was 0.999 (95% CI: 0.996–1.000), and the test-set AUC was 0.769 (95% CI: 0.611–0.928), indicating that the model had good discriminative ability for the two outcomes of ovarian preservation/non-preservation, providing a reliable model basis for subsequent interpretability analysis.

#### 3.5.2 Global feature importance: ranking and direction of action of core influencing factors

Through an analysis of the SHAP feature importance bar plots (training and test sets), SHAP summary plots, and SHAP feature distribution violin plots, the core features affecting ovarian preservation prediction and their action patterns were highly stable between the training and test sets. Among core factors affecting ovarian preservation, height was the most critical factor, with mean absolute SHAP values of 0.05 and 0.04 in the training and test sets, respectively. In Figure 6F, the higher height range corresponded with more concentrated positive SHAP values; the training-set summary plot (Figure 6B) showed that SHAP values were mostly positive when height was in the higher range, which could increase the probability of ovarian preservation and simultaneously reflect the enhancement of ovarian blood supply and repair potential in post-pubertal children. The trend of SHAP value distribution of height in the test-set summary plot (Figure 6D) was consistent with that in the training-set summary plot, further verifying its core driving role in ovarian preservation. Hb ranked second, with mean absolute SHAP values of 0.04 in both the training and test sets. In Figure 6F, the normal/higher Hb level corresponded to a higher proportion of positive SHAP values; the summary plot showed that SHAP values were mostly positive when Hb was at normal or higher levels, and sufficient oxygen supply was an important basis for ovarian preservation. When Hb was low, SHAP values were negative, and insufficient tissue oxygen supply at this time would significantly increase the risk of ovarian IN, which was consistent with the pathological mechanism that tissue oxygen supply after torsion determines survival potential. The CRP and ultrasound signals constituted the third-level core features. Among these features, the mean absolute SHAP values of CRP in the training and test sets were 0.04 and 0.03, respectively. In Figure 6F, the proportion of positive SHAP values in the lower level range of CRP was higher. The summary plot showed that CRP mostly had a positive contribution when it was low, indicating that ovarian tissue still maintained a certain degree of activity under mild inflammatory conditions; when the CRP level was high, it turned to a negative contribution, indicating that severe inflammation often indicates irreversible IN of ovarian tissue. The mean absolute SHAP value of the ultrasonic signal in the test-set bar plot (Figure 6E) reached 0.04, and both Figure 6F and the summary plot showed that ultrasonic manifestations with features related to ovarian preservation could significantly increase the predicted probability of ovarian preservation, thereby directly verifying the key value of ultrasound in evaluating ovarian viability.

#### 3.5.3 Local interpretation of single-sample prediction: analysis of individual contributions by SHAP force plot

Taking a sample from the test set as an example (Figure 6G), the baseline predicted probability of the model was approximately 0.20, and the final predicted probability was 0.21. The force plot intuitively presents the promoting effect of each feature on the prediction result through color and line length.

- Red segments: lymphocyte count (LYMPH) = 2.59; CRP = 18.94 (this value was in the “mildly elevated but not reaching the severe necrosis threshold” range); ultrasonic signal (Ultrasound Signal) = 2.0 (this signal indicated that the ovary had ultrasonic manifestations related to preservation). These features promoted an increase in the predicted probability through the red-line segments, reflecting their potential for ovarian preservation.
- Blue segments: Hb = 119.0 (this value was slightly lower than the lower limit of the normal reference value, indicating insufficient tissue oxygen supply); height = 1.61 (not in the “high height advantage range,” reflecting limited ovarian development potential); affected/unaffected side (Affected_Unaffected) = 3.4 (indicating a wide range of lesion involvement); SII = 1,160.764479 (indicating imbalanced inflammatory response); vascular signal (Vascularity Signal) = 0.0 (this signal directly indicated poor ovarian blood supply). These features reduced the probability predicted through the blue line segments, weakening the possibility of ovarian preservation.

The final predicted probability of this sample was consistent with the clinical evaluation of high difficulty in ovarian preservation, which intuitively showed the combined impact of multi-dimensional factors (inflammatory status, ultrasonic manifestations, basic physiological indicators) on ovarian preservation, verifying the interpretability and accuracy of the model at the individual level.

## 4 Discussion

### 4.1 Correspondence between core results and clinical value

The ML model (OVART-ML) constructed in this study showed excellent performance in predicting the risks of OT and IN in children: the test-set AUC of the SVM model for OT was 0.911 (95% CI: 0.809–1.000), and the test-set AUC for ovarian preservation was 0.769 (95% CI: 0.611–0.928). SHAP analysis identified eight key influencing factors: the follicular edema ring sign, vomiting, pelvic effusion, EOS, WBC, Hb, NER, and SII. Among these, the follicular edema ring sign (mean |SHAP value| = 0.12) and EOS (mean |SHAP value| = 0.08) were the core driving factors for torsion prediction, whereas height (mean |SHAP value| = 0.05) and Hb (mean |SHAP value| = 0.04) were the key indicators for ovarian preservation. These results are highly consistent with clinical, pathological, and physiological mechanisms: the follicular edema ring sign is a specific ultrasonic manifestation of early congestion and edema in OT (16), decreased EOS reflects an immune imbalance caused by ischemia-reperfusion injury (IRI) after torsion (17, 18), and Hb maintains ovarian tissue oxygen supply to prevent IN (19). These findings provide a quantitative basis for the entire clinical process from acute diagnosis to postoperative preservation decision-making. Another strength of this study lies in identifying a classification threshold that balances statistical merit and clinical utility. The optimal threshold (0.669), determined based on Youden's index, allows the model to achieve both high sensitivity (0.9000) and high specificity (0.8571, Youden's index = 0.7571). As shown in Table 3, we further developed a “threshold-strategy framework” to provide decision-making flexibility for diverse clinical scenarios: for emergency screening, a low threshold (e.g., 0.2) can be adopted to leverage the advantage of high sensitivity for ruling out the diagnosis; for preoperative evaluation, a high threshold (e.g., 0.8) can be used to utilize the high specificity for aiding in confirming the diagnosis. This flexibility significantly enhances the applicability of the OVART-ML model in real-world clinical settings.

### 4.2 Innovations and breakthroughs of this study

Compared with existing studies, this study achieved three breakthroughs in methodology, clinical coverage, and interpretability. First, a dual-outcome prediction model was constructed. Existing studies have mostly focused on a single target; for example, Asya et al. (20) only predicted pediatric adnexal torsion without involving ovarian preservation, while Ahmad et al. (4) only differentiated appendicitis and lacked universality. This study integrated the dual outcomes of torsion occurrence and ischemic necrosis, filling the gap in tools that connect diagnosis and prognosis.

Second, in-depth integration of multimodal data was achieved. Previous studies have mostly relied on single-type data [some studies only used ultrasound (16), which is prone to missed diagnoses, while others relied only on physical signs and simple laboratory results (21, 22), which are difficult to differentiate]. This study integrated 27 variables into four categories, including derived indicators such as SII (23) and NER (24), and the AUC increased by 7.8%–24.6% (25, 26) compared with single-type models, significantly improving the ability to identify atypical cases.

Third, model interpretability and pediatric applicability were enhanced. ML is limited by its black-box characteristics (27). This study achieved a triple interpretation (global: feature importance; local: single-sample logic; and interaction: feature synergy) through SHAP, enabling the model to explain the underlying reasons. The pathology of torsion in children is significantly different from that in adults (28), and the existing adult studies (21) are not applicable. This study targeted 112 children, screened for specific indicators such as height, verified its core impact on ovarian preservation, and solved the adaptability problem of applying adult models to pediatrics.

### 4.3 Analysis of multi-dimensional predictive factors for ovarian torsion

Through model analysis, this study identified multidimensional predictive factors for pediatric OT from the perspectives of ultrasonic features, symptom-imaging combinations, and laboratory indicators, providing a basis for early clinical identification. As the core ultrasonic feature, the follicular edema ring sign had the highest mean absolute SHAP value in the SHAP analysis and remained the primary predictive factor in both training and test sets. The overall detection rate was 40.2%, with 41.6% in the ovarian preservation group and 34.8% in the necrosis group (Table 1). This sign is a specific manifestation of early congestion and edema caused by impaired venous return after OT. The thin ovarian capsule in children makes the edema ring more easily identifiable on ultrasound (29). Therefore, in clinical practice, when this sign is detected by ultrasound, the predicted probability of OT exceeds 60% (positive SHAP contribution: 0.10–0.15) even if the child only presents with mild abdominal pain (without vomiting), and prompt completion of EOS and WBC tests is required. If combined with EOS < 0.05 × 10^9^ /L (median EOS in the necrosis group: 0.03 × 10^9^ /L), the synergistic effect of these two factors increases the OT probability to over 80% (positive SHAP contribution: 0.15–0.20), and emergency laparoscopic exploration should be initiated. In cases where this sign is detected alone but EOS is within the normal range (>0.1 × 10^9^ /L, median EOS in the preservation group: 0.09 × 10^9^ /L), the OT probability decreases to below 40%, and short-term dynamic observation is feasible.

Vomiting + pelvic effusion as an early warning signal for torsion in young children, SHAP analysis showed that the mean |SHAP value| of vomiting was 0.05 and that of pelvic effusion was 0.04. The incidence of vomiting in the necrosis group was 52.2% (higher than 40.4% in the preservation group), and the incidence of pelvic effusion was 56.5% (higher than 46.1% in the preservation group) (Table 1). The SHAP interaction plot showed that when vomiting was 1, the contribution of pelvic effusion to torsion prediction doubled. Vomiting is a typical manifestation of peritoneal irritation caused by torsion in children [young children often cannot express abdominal pain; therefore, vomiting has a higher specificity (30, 31)], and pelvic effusion is the result of abdominal exudation secondary to torsion (the amount of effusion is positively correlated with the duration of torsion). A combination of the two can compensate for the diagnostic deficiency caused by the lack of chief complaints in young children (32). Therefore, in clinical practice, for children aged <5 years who cannot accurately report abdominal pain, if vomiting (especially projectile vomiting) and pelvic effusion coexist, even if the follicular edema ring sign is not detected, the torsion probability exceeds 50%, and emergency ultrasonic Doppler examination (to evaluate ovarian blood flow signals) should be performed. For children with vomiting and pelvic effusion but EOS > 0.1 × 10^9^ /L and WBC < 10 × 10^9^ /L (median WBC in the preservation group: 8.33 × 10^9^ /L), mild torsion is mostly indicated, and conservative treatment (e.g., fasting, fluid replacement) can be attempted; if vomiting persists for >6 h and the amount of pelvic effusion increases, even if EOS is normal, surgical exploration is required.

EOS and SII are key laboratory indicators. The mean |SHAP value| of EOS was 0.08 (ranking second). Table 1 shows that the median EOS in the necrosis group was 0.03 × 10^9^ /L (significantly lower than 0.09 × 10^9^ /L in the preservation group). The SHAP dependence plot showed that when EOS < 0.2 × 10^9^ /L, SHAP values decreased rapidly with decreasing EOS. IRI after torsion activates the immune cell apoptosis pathway, and EOS is more sensitive to ischemia, leading to a decrease in peripheral blood count, accompanied by increased neutrophil chemotaxis and resulting in an inflammatory imbalance (33, 34). Therefore, in clinical practice, EOS < 0.03 × 10^9^ /L indicates the possibility of torsion; if combined with SII > 1,500 (median SII in the necrosis group: 1,524.2), the risk of torsion should be alerted. For EOS > 0.1 × 10^9^ /L, even if pelvic effusion exists, the torsion probability is still <30%, and conservative observation should be prioritized. The mean |SHAP value| for the SII was 0.04. Table 1 shows that the median SII in the necrosis group was 1,524.2 (2.7 times that of the preservation group: 573.9). The SHAP dependence plot shows that for SII < 1,000, the SHAP values increased rapidly with increasing SII. The SII integrates platelets (procoagulant), neutrophils (anti-inflammatory), and lymphocytes (immune), and comprehensively reflects the systemic inflammatory state after torsion, which is positively correlated with the severity of IRI (35). Therefore, in clinical practice, SII > 1,500 and CRP > 10 mg/L (median CRP in the necrosis group: 18.17 mg/L) indicate that inflammation has involved the ovarian parenchyma; SII < 500 and CRP < 5 mg/L (median CRP in the preservation group: 0.27 mg/L) indicate a low risk of IN. When SII is in the range of 1,000–1,500, the risk level should be determined in combination with EOS (EOS < 0.05 × 10^9^ /L indicates high risk, and EOS > 0.05 × 10^9^ /L indicates medium risk).

By integrating multidimensional factors (ultrasound, symptom imaging, and laboratory findings), the model clarified the core role of the follicular edema ring sign, vomiting + pelvic effusion, EOS, and SII in torsion identification. These factors complement each other, significantly improving the accuracy of identifying typical and atypical cases and providing a coherent judgment system for clinical torsion diagnosis.

### 4.4 Exploration of key influencing factors for ovarian ischemic necrosis risk

Through further model analysis, this study revealed the key impacts of laboratory-derived and physiological indicators on the risk of ovarian IN, providing a basis for evaluating the tissue survival potential and formulating treatment decisions.

#### 4.4.1 Early warning value of EOS for necrosis risk

The mean |SHAP value| was 0.08 (ranking second). Table 1 shows that the median EOS in the necrosis group was 0.03 × 10^9^ /L (significantly lower than 0.09 × 10^9^ /L in the preservation group). IRI after torsion activates the immune cell apoptosis pathway, and EOS is more sensitive to ischemia, leading to a decrease in peripheral blood count accompanied by increased neutrophil chemotaxis and resulting in an inflammatory imbalance. This imbalance is directly related to the irreversibility of necrosis (33, 34). Therefore, in clinical practice, EOS < 0.03 × 10^9^ /L not only indicates torsion but also predicts an increased risk of IN. If EOS < 0.03 × 10^9^ /L is combined with SII > 1,500 (median SII in the necrosis group: 1,524.2), the necrosis probability exceeds 50%, and ovarian viability should be focused on during surgery (e.g., evaluating incision bleeding, color changes); if EOS remains <0.05 × 10^9^ /L after surgery, occult ischemia may exist, and anti-inflammatory treatment should be strengthened (36, 37).

#### 4.4.2 NER as a judgment indicator for necrosis irreversibility

The mean |SHAP value| was 0.01. Table 1 shows that the median NER in the necrosis group was 307.3 (six times that of the preservation group: 49.25). The SHAP dependence plot revealed that, when NER was <200, the SHAP values increased rapidly with increasing NER. After torsion, increased neutrophil chemotaxis (anti-inflammatory response) and decreased EOS (immune suppression) lead to elevated NER, indicating a more severe inflammatory imbalance dominated by relative neutrophil predominance and more irreversible damage to the ovarian parenchyma, which is highly consistent with the pathological findings of necrosis (38, 39). Therefore, in clinical practice, when NER is >200, even if the ovary does not show obvious darkening in appearance, occult necrosis should be alerted, and intraoperative frozen section pathology examination is recommended; if the pathological result indicates a high rate of parenchymal cell necrosis, oophorectomy should be performed decisively. When NER is <100, the probability of ovarian preservation increases significantly, and even if torsion lasts for >6 h, detorsion surgery can be attempted.

#### 4.4.3 Hb as a predictor of ovarian repair potential in children

The mean |SHAP value| of height was 0.05 (ranking first in ovarian preservation prediction), and that of Hb was 0.04. Table 1 shows that the median height in the preservation group was 115 cm (greater than 84 cm in the necrosis group), and the median Hb was 130.5 g/L (greater than 125 g/L in the necrosis group). The SHAP summary plot indicated that the SHAP values were mostly positive when height was >130 cm and Hb was >120 g/L. Height is positively correlated with the degree of ovarian development in children (children with height >130 cm are mostly post-pubertal, with relatively richer ovarian blood supply and stronger vascular repair potential); Hb reflects tissue oxygen supply: Hb > 120 g/L can mostly ensure sufficient oxygen supply to the ovary after detorsion, promoting parenchymal cell repair and reducing the risk of necrosis (40). Therefore, in clinical practice, for children with a height of >130 cm (postpubertal) and Hb level >120 g/L, the probability of ovarian preservation is significantly higher, and detorsion surgery should be prioritized. For children with height <110 cm (preschool age) and Hb < 110 g/L, the preservation probability decreases significantly; if the ovary appears pale and bloodless during surgery, direct OPE can be performed. For children with heights in the range of 110–130 cm and Hb in the range of 110–120 g/L, detorsion can be attempted first. Ultrasonic blood flow re-examination should be conducted 48 h postoperatively; if no blood flow signal is still detected, a second surgical resection can be performed.

#### 4.4.4 Stratifying the role of SII in the severity of necrosis-related inflammation

The mean |SHAP value| was 0.04. Table 1 shows that the median SII in the necrosis group was 1,524.2 (2.7 times that of the preservation group: 573.9). The SHAP dependence plot shows that when SII < 1,000, the SHAP values increased rapidly with increasing SII. The SII integrates platelets, neutrophils, and lymphocytes and comprehensively reflects the systemic inflammatory state after torsion, which is positively correlated with the severity of IRI. It can quantify the degree of inflammatory damage to the ovarian parenchyma (35). Therefore, in clinical practice, SII > 1,500 combined with CRP > 10 mg/L (median CRP in the necrosis group: 18.17 mg/L) indicates that inflammation involves the ovarian parenchyma, and anti-inflammatory treatment should be strengthened postoperatively. SII < 500 combined with CRP < 5 mg/L (median CRP in the preservation group: 0.27 mg/L) indicates a low risk of ovarian necrosis even if torsion exists, and no excessive intervention is needed postoperatively. If the preoperative SII is in the range of 1,000–1,500, the risk level should be determined in combination with the EOS to guide treatment.

Together, this model clarified the core values of EOS, NER, height + Hb, and SII for the assessment of ovarian IN. These factors reveal the risk of necrosis from the perspective of inflammatory imbalance, tissue repair potential, and inflammatory severity, providing precise references for clinicians to formulate ovarian preservation strategies and optimize treatment plans.

### 4.5 Study limitations and future prospects

This study has several limitations. First, it is a retrospective, two-center study that included only 112 patients, with the validation set consisting of merely 34 cases. This relatively small sample size limits the model's statistical power, increases the risk of overfitting, and may compromise its external validity and generalizability. The retrospective design itself also makes it difficult to avoid selection bias—for instance, patients with more typical or severe conditions may be more likely to be included in the analysis.

Second, we must be cautious about the risk of “threshold overfitting”. As elaborated by Arredondo Montero, the optimal diagnostic threshold identified through optimization (e.g., Youden's index) on a single dataset may degrade in performance when applied to new populations. Although we employed training-test set split and cross-validation, the probability threshold used in our model still needs to be validated and potentially calibrated in larger-scale, prospective cohorts to ensure the robustness of its clinical decision-making. Additionally, this study has potential verification bias. The diagnosis of ovarian IN relies on postoperative pathological examination, and only cases that underwent oophorectomy can obtain pathological confirmation. For ovaries preserved intraoperatively, the true pathological status cannot be evaluated, and this discrepancy may introduce estimation bias. Meanwhile, the outcome of ovarian preservation was only based on intraoperative evaluation and pathological diagnosis, and long-term follow-up data [e.g., ovarian hormone levels and follicular development (41)] at 6–12 months postoperatively were not included; therefore, the normal physiological function of the preserved ovary could not be verified. Future research plans aim to directly address these limitations. We intend to conduct a prospective multicenter cohort study enrolling 500 patients, with the incorporation of an external validation set. Additionally, we will develop an online calculator or mobile application to dynamically evaluate and optimize the decision threshold in real-world clinical settings. Furthermore, ultrasonic radiomic features (42) and metabolomic indicators will be introduced to construct a clinical-imaging-omics multidimensional model, enhancing the ability to identify atypical torsion and occult necrosis.

## 5 Conclusion

The OVART-ML model constructed in this study innovatively realizes dual-outcome prediction of OT and IN risks in children. Through multimodal data integration and SHAP interpretability analysis, key influencing factors such as the follicular edema ring sign, EOS, height, and Hb level were identified. The model exhibited excellent performance and aligned with clinical needs. This study not only fills the gap in tools for continuous prediction of pediatric OT-ovarian preservation but also breaks the black box barrier of ML through interpretability design, providing pediatricians with a decision-support tool that combines accuracy and practicality. This model is expected to be incorporated into routine clinical assessment processes after multicenter validation, thereby contributing to the precise protection of ovarian function in children.

## Data Availability

The raw data supporting the conclusions of this article will be made available by the authors, without undue reservation.
