# Supplementary material for: Construction and validation of a machine learning model integrating ultrasound features and inflammatory markers (OVART-ML) for predicting ovarian torsion and ischemic necrosis risk in children
Source: Front Pediatr. 2025 Dec 4;13:1717545. doi: 10.3389/fped.2025.1717545 (PMC12711762; doi:10.3389/fped.2025.1717545)
Supplement: Supplementary file 1 [file Table1.docx]

**Suppl.Table 1|** Bootstrap Internal Validation Results for Ovarian Torsion Prediction Models (Test Set: n=34, Events=20)​​

| Model | AUC (Original) | AUC (Corrected) | Optimism | Brier Score (Original) | Brier Score (Corrected) | Optimism | Overfitting Degree |
| --- | --- | --- | --- | --- | --- | --- | --- |
| LR | 0.889 | 0.883 | 0.006 | 0.095 | 0.090 | 0.005 | Mild |
| NB | 0.886 | 0.884 | 0.002 | 0.090 | 0.091 | -0.001 | Mild |
| ​​SVM​​ | ​​0.911​​ | ​​0.907​​ | ​​0.003​​ | ​​0.120​​ | ​​0.124​​ | ​​-0.003​​ | ​​Mild​​ |
| KNN | 0.912 | 0.918 | -0.005 | 0.095 | 0.096 | -0.000 | Mild |
| RF | 0.814 | 0.813 | 0.002 | 0.172 | 0.174 | -0.002 | Mild |
| ET | 0.930 | 0.933 | -0.003 | 0.094 | 0.092 | 0.002 | Mild |
| XGB | 0.887 | 0.891 | -0.004 | 0.109 | 0.113 | -0.004 | Mild |
| LGB | 0.904 | 0.901 | 0.003 | 0.129 | 0.126 | 0.003 | Mild |
| GB | 0.827 | 0.826 | 0.000 | 0.158 | 0.157 | 0.000 | Mild |
| AdaBoost | 0.821 | 0.822 | -0.001 | 0.211 | 0.213 | -0.001 | Mild |
| MLP | 0.886 | 0.883 | 0.002 | 0.117 | 0.116 | 0.001 | Mild |
