# Supplementary material for: Construction and validation of a machine learning model integrating ultrasound features and inflammatory markers (OVART-ML) for predicting ovarian torsion and ischemic necrosis risk in children
Source: Front Pediatr. 2025 Dec 4;13:1717545. doi: 10.3389/fped.2025.1717545 (PMC12711762; doi:10.3389/fped.2025.1717545)
Supplement: Supplementary file 2 [file Supplementaryfile1.docx]

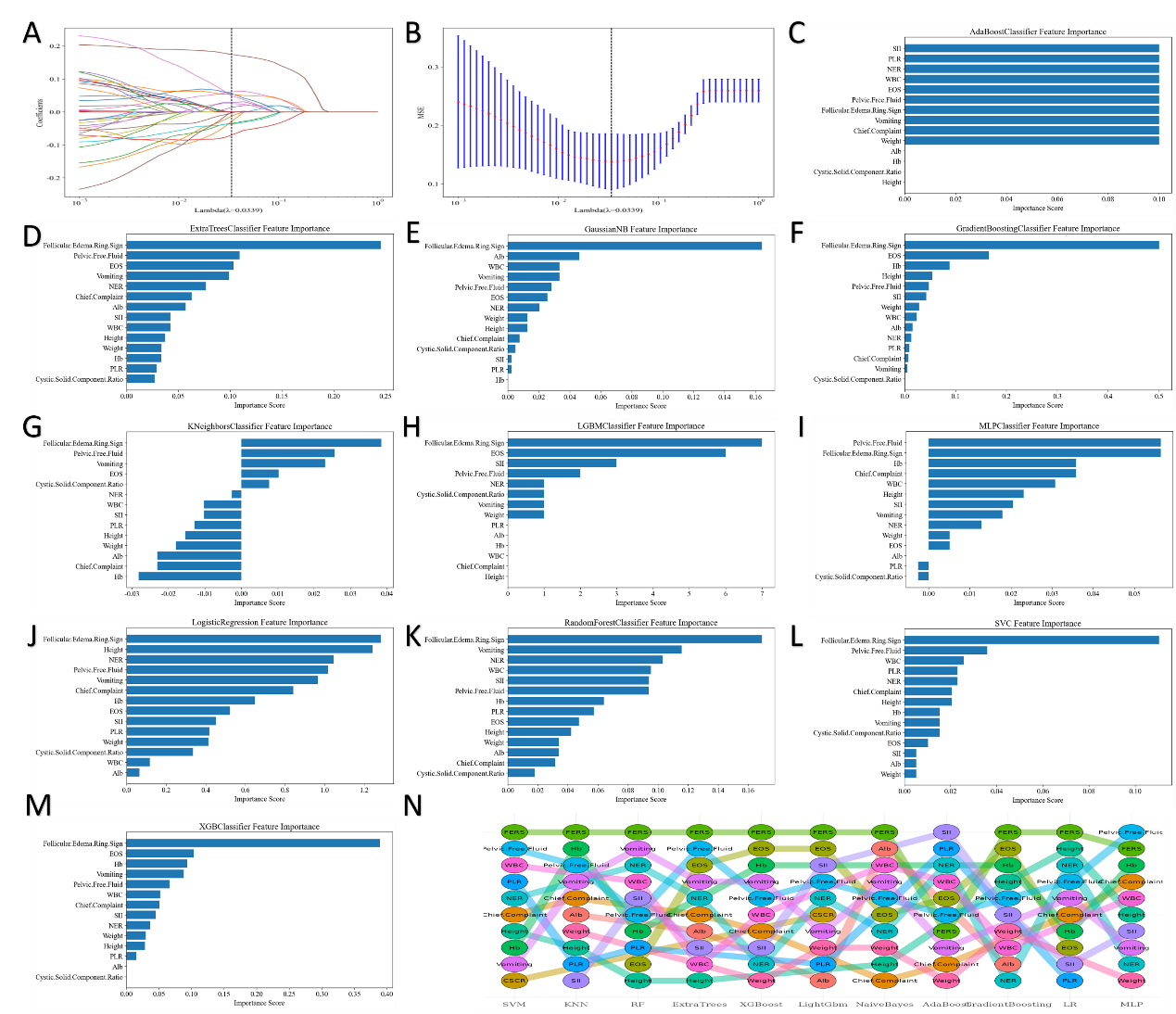


**Suppl. Fig. 1|** LASSO coefficient path plot: Demonstrates how the magnitude of feature coefficients evolves with changes in the regularization parameter. (A) LASSO cross-validation (CV) plot: Illustrates the association between λ values and the mean cross-validated error, where the dashed line marks the optimal λ.(B) Feature importance bar plots for different classifiers: Each subplot ranks features by their importance scores in the corresponding model(C-M): AdaBoostC), ExtraTrees(D), Gaussian Naive Bayes(E), GradientBoosting(F), KNeighbors(G), LGBM(H), MLP(I), LogisticRegression(J), RandomForest(K), SVC(L), XGBoost(M). MLP network structure visualization(N).


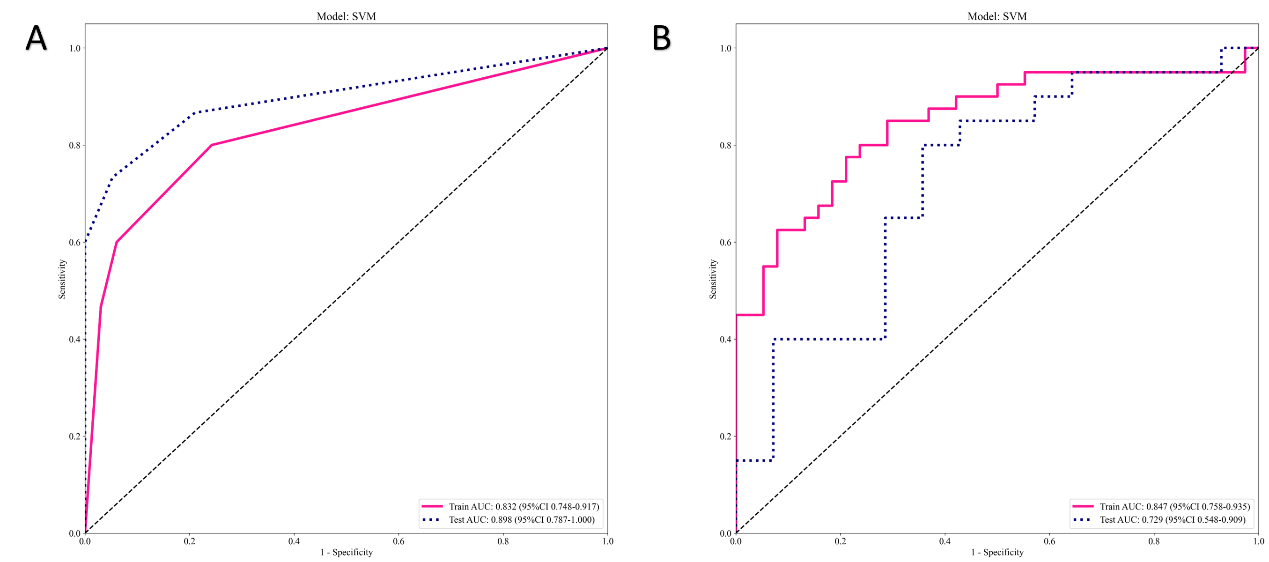


**Suppl. Fig. 2|** ROC curve of SVM model with ultrasound features only. (A) ROC curve of SVM model with serological indices only. (B)


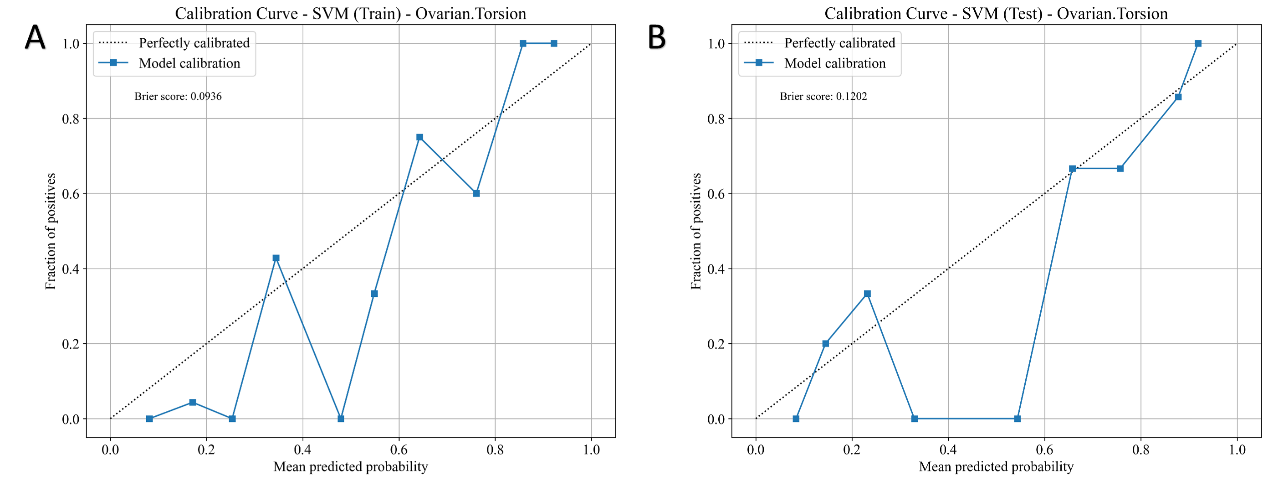


**Suppl. Fig. 3|** Calibration curve of SVM model on ovarian torsion training set. (A) Calibration curve of SVM model on ovarian torsion test set. (B)
