# Supplementary material for: Construction and validation of a machine learning model integrating ultrasound features and inflammatory markers (OVART-ML) for predicting ovarian torsion and ischemic necrosis risk in children
Source: Front Pediatr. 2025 Dec 4;13:1717545. doi: 10.3389/fped.2025.1717545 (PMC12711762; doi:10.3389/fped.2025.1717545)
Supplement: Supplementary file 3 [file Supplementaryfile2.docx]

**Hyperparameter Optimization and Model Training**

Hyperparameter optimization and model training were conducted to identify the optimal model configuration for predicting ovarian torsion (OT) and ovarian ischemic necrosis (IN). Specifically, grid search (GridSearchCV) combined with 5-fold cross-validation was employed to systematically tune the hyperparameters of 11 machine learning algorithms.For support vector machines (SVM), hyperparameter search was performed under two kernel functions: linear kernel (penalty parameter C∈{0.1,1,10,100}) and radial basis function (RBF) kernel (C∈{0.1,1,10,100},  γ∈{0.001,0.0001}). For k-nearest neighbors (KNN) models, combinatorial optimization was applied to the number of neighbors (n_neighbors∈{3,5,7,9}), weight strategy (uniform or distance), and distance metric ( p = 1 or 2). Both random forest and extra trees models explored hyperparameters including the number of trees (n_estimators∈{50,100,200}), maximum tree depth (max_depth∈{None,10,20,30}), minimum samples required to split an internal node (min_samples_split∈{2,5,10}), and minimum samples required at a leaf node (min_samples_leaf∈{1,2,4}).For gradient boosting-based models, the tuned hyperparameters of XGBoost and LightGBM included the number of trees (50–200), learning rate (0.01–0.2), tree depth (XGBoost: 3–9; LightGBM: number of leaf nodes 31–127), subsample rate (subsample∈{0.8,1.0}), and feature sampling ratio (colsample_bytree∈{0.8,1.0}). For AdaBoost and gradient boosting trees (GradientBoosting), the primary optimized hyperparameters were the number of iterations (50–200) and learning rate (0.01–1.0); the latter additionally included maximum depth (3–7) and subsample rate. Logistic regression models were tested with different regularization strengths (C∈{0.001,0.01,0.1,1,10,100}) under L1 or L2 regularization, with the solver fixed as liblinear. For multilayer perceptrons (MLP), combinatorial search was performed on hidden layer structures ((50,), (100,), (50,50)), activation functions (tanh or relu), optimizers (sgd or adam), L2 regularization coefficients (α∈{0.0001,0.05}), and learning rate scheduling strategies (constant or adaptive).Naive Bayes was included directly as a baseline for comparison, as it lacks tunable hyperparameters. Notably, for XGBoost and LightGBM, the early stopping mechanism was enabled during training (early_stopping_rounds=50), with the AUC of the validation set used as the monitoring metric. Training was terminated early if no improvement was observed for 50 consecutive rounds, to prevent overfitting and improve computational efficiency. All tuning processes were implemented in the Python 3.8 environment using open-source libraries including scikit-learn, XGBoost, and LightGBM.
